# Potential Use of L-arabinose for the Control of Tomato Bacterial Wilt

**DOI:** 10.1264/jsme2.ME20106

**Published:** 2020-10-22

**Authors:** Hui-Zhen Fu, Malek Marian, Takuo Enomoto, Haruhisa Suga, Masafumi Shimizu

**Affiliations:** 1 The United Graduate School of Agricultural Science, Gifu University, 1–1 Yanagido, Gifu, Gifu 501–1193, Japan; 2 Faculty of Applied Biological Sciences, Gifu University, 1–1 Yanagido, Gifu, Gifu 501–1193, Japan; 3 College of Agriculture, Ibaraki University, 3–21–1 Chuuo, Ami, Inashiki, Ibaraki 300–0393, Japan; 4 Life Science Research Center, Gifu University, 1–1 Yanagido, Gifu, Gifu 501–1193, Japan

**Keywords:** L-arabinose, tomato, bacterial wilt, soil drenching, *Ralstonia pseudosolanacearum*

## Abstract

The present study aimed to investigate the potential of simple sugars for use as protection agents in the control of tomato bacterial wilt caused by *Ralstonia pseudosolanacearum*. Based on the sugar assimilation patterns of the pathogen, four unassimilable sugars (L-arabinose, maltose, D-raffinose, and D-ribose) were selected from 10 representative sugars present in tomato root exudates. These sugars were evaluated for their effects on bacterial wilt using a tomato seedling bioassay. The application of 0.25% L-arabinose significantly reduced disease severity and was, thus, selected as a candidate for further evaluations in a pot experiment under glasshouse conditions. The results obtained showed that the disease suppressive effects of L-arabinose slightly increased at higher concentrations; drench treatments at 0.1, 0.25, and 0.5% reduced disease severity by *ca.* 48, 70, and 87%, respectively. The drench treatment with 0.5% L-arabinose significantly reduced the pathogen population in the rhizosphere and stem tissues of tomato plants without any antibacterial activity. Real-time reverse-transcription PCR revealed that the expression of salicylic acid-dependent and ethylene-dependent defense genes was significantly enhanced in the stem tissues of L-arabinose-treated tomato plants following the pathogen inoculation. These results suggest that soil drenching with L-arabinose effectively suppresses tomato bacterial wilt by preventing pathogen proliferation in the rhizosphere and stem tissues of tomato plants. This is the first study to report the potential of L-arabinose as a safe, eco-friendly, and cost-effective plant protection agent for the control of tomato bacterial wilt.

Plant diseases caused by bacterial pathogens represent a considerable yield and quality constraint for farmers worldwide. The *Ralstonia solanacearum* species complex (RSSC), which includes *R. solanacearum*, *Ralstonia pseudosolanacearum*, and *Ralstonia syzygii*, is regarded as one of the most important soil-borne, plant pathogenic bacterial groups (Safni *et al.*, 2014). RSSC infects more than 250 plant species in approximately 50 families and causes extensive yield losses in economically important crops, particularly solanaceous plants, such as potato, tomato, pepper, and eggplant (Peeters *et al.*, 2013).

Tomato (*Solanum lycopersicum* L.) is one of the most valuable vegetable crops worldwide. According to data from the Food and Agriculture Organization, tomato is cultivated in more than 170 countries, accounting for a total harvest area of approximately 4.8 million hectares and produce of approximately 182 million tons in 2018 (FAOSTAT, 2018). Bacterial wilt, caused by *R. solanacearum* and *R. pseudosolanacearum*, is one of the major constraints in tomato production in tropical and subtropical climates with plant mortality up to 90% (Yuliar *et al.*, 2015). Until now, various strategies have been suggested for the control of tomato bacterial wilt, including soil fumigation (Enfinger *et al.*, 1979; Ji *et al.*, 2005) and the use of grafted tomatoes (Rivard *et al.*, 2012). However, these control measures have achieved limited success because of the causal pathogen’s wide host range, high genetic variability, and ability to survive in deep soil layers (Sahu *et al.*, 2017). In addition, the chemicals used to fumigate soil have adverse or even harmful effects in humans, in animals, and on the environment and, as such, the use of chemical fumigants needs to be minimized as much as possible (Suchoff *et al.*, 2019). Therefore, the development of reliable, cost-effective, and eco-friendly approaches for the control of tomato bacterial wilt is urgently needed.

The use of natural bioactive compounds derived from plants and animals has recently been proposed as a new and attractive approach for plant disease control and an important component of an integrated disease management program (Jamiołkowska, 2020). Many types of antimicrobial phenolics, terpenoids, saponins, coumarins, and alkaloids have been discovered in plants, and some have been reported to have the capacity to suppress fungal and bacterial diseases, including bacterial wilt (Lattanzio *et al.*, 2006; Gurjar *et al.*, 2012; Seo *et al.*, 2012; Yuan *et al.*, 2012; Shad *et al.*, 2014; Yang *et al.*, 2018). In addition, several non-antimicrobial substances, such as polysaccharides, amino acids, and sugars, have been identified as potential plant protection agents for bacterial wilt control (Algam *et al.*, 2010; Posas and Toyota, 2010; Kiirika *et al.*, 2013). Posas *et al.* (2007) previously reported that a soil treatment with glucose, proline, glutamine, serine, arginine, and lysine effectively suppressed tomato bacterial wilt, possibly by enhancing soil microbial activity. Moreover, Seo *et al.* (2016) recently demonstrated that the application of L-histidine to roots activated ethylene-mediated defense responses and inhibited bacterial wilt in tomato and *Arabidopsis* plants. Among these natural organic compounds, sugars are the most abundant in the biosphere, are relatively cheap, and are commonly used in foods and beverages (Cummings and Stephen, 2007; Goldfein and Slavin, 2015). Therefore, they are regarded as a good source of safe, eco-friendly, and economical plant protection agents.

Thus, the objective of the present study was to examine the potential of simple sugars released by tomato roots in the control of tomato bacterial wilt and to investigate potential suppressive mechanisms.

## Materials and Methods

### Pathogen

*R. pseudosolanacearum* strain VT0801 (Marian *et al.*, 2018) was used as the challenging pathogen. VT0801 was pre-cultured on a casamino acid-peptone-glucose (CPG) agar plate (Kelman, 1954) at 30°C for 48 h. After the incubation, bacterial cells harvested from the CPG agar plate were inoculated into fresh CPG broth and cultured at 30°C for 24 h with shaking at 200 rpm.

### Plant materials

The susceptible tomato cultivar ‘Ponderosa’ (*S. lycopersicum* L.) was used in the present study. Seeds were surface sterilized in 70% (v/v) ethanol for 1‍ ‍min and 2% (v/v) sodium hypochlorite for 5‍ ‍min, followed by rinsing six times with sterile distilled water (SDW). Sterilized seeds were germinated at 25°C in the dark for 3 days on filter paper moistened with SDW.

### Sugar assimilation by *R. pseudosolanacearum*

In the present study, we screened sugars released from tomato roots for potential use as chemical control agents against tomato bacterial wilt because they may be less harmful to tomato plants. However, we speculated that the application of sugars assimilated by *R. pseudosolanacearum* as a source of energy may increase the incidence of bacterial wilt disease. Therefore, we selected sugars unassimilable to *R. pseudosolanacearum* from a list of 10 sugars (*viz.*, L-arabinose, D-fructose, D-galactose, D-glucose, maltose, D-mannose, D-raffinose, D-ribose, sucrose, and D-xylose) released from tomato roots (Huang *et al.*, 2017; Suarez-Fernandez *et al.*, 2020 Chitosan induces plant hormones and defences in tomato root exudates. *bioRxiv* doi.org/10.1101/2020.06.09.142653). The ability of strain VT0801 to assimilate these 10 sugars was examined as described below. The cells of strain VT0801 were harvested from the above 24-h-old CPG culture by centrifugation at 9,900×*g* for 10‍ ‍min, washed in 1/4 strength M63 medium broth (without 0.2% glycerol) (Amresco), and suspended in 1/4 strength M63 medium broth (without 0.2% glycerol). The concentration of the cell suspension was adjusted to an optical density at 600 nm (OD_600_) of 0.1 (approximately 9×10^7^ CFU mL^–1^). A 10-μL aliquot of the cell suspension was then inoculated into test tubes (15×105‍ ‍mm, Pyrex Test-Tube/P-15s; Nichiden-Rika Glass) containing 3‍ ‍mL of 1/4 strength M63 medium broth supplemented with each sugar as a sole carbon source at a final concentration of 0.25% (w/v). The tube containing non-supplemented 1/4 strength M63 medium broth was inoculated with strain VT0801 and served as a control. These tubes were incubated at 30°C for 48 h with shaking at 200 rpm. Bacterial growth was assessed by measuring the OD_600_ of the culture broth using a spectrophotometer (GeneQuant pro Spectrophotometer, Amersham Biosciences). Three replicate tubes were used for each sugar.

### Tomato seedling bioassay

Sugars that were not assimilated by strain VT0801 in the above experiment were evaluated for their effects on bacterial wilt using a tomato seedling bioassay (Marian *et al.*, 2018) with a slight modification. As shown in Fig. S1, five germinated tomato seeds were sown into half of the surface of an autoclaved vermiculite layer (3.2 g) in a flat-bottomed glass tube (25×125‍ ‍mm; AGC Techno Glass). After sowing, a 2-mL aliquot of a 0.25% (w/v) solution of each sugar (dissolved in SDW) was applied to the vermiculite layer in the tube. The control treatment was prepared using 2‍ ‍mL of SDW instead of the sugars. The seeds were then covered with approximately 0.2‍ ‍g of autoclaved vermiculite and maintained in a controlled environmental chamber (Biotron, standard; Nippon Medical and Chemical Instruments) at 28°C under a 12-h light/dark cycle. The cells of strain VT0801 were harvested from the 24-‍h CPG culture broth by centrifugation at 9,900×*g* for 10‍ ‍min, washed with sterile 10‍ ‍mM MgCl_2_·6H_2_O, and suspended to *ca.* 9×10^5^ CFU mL^–1^ in sterile 10‍ ‍mM MgCl_2_·6H_2_O. On day 3 after sowing, the cell suspension of strain VT0801 was inoculated into the area opposite to where the tomato seedlings grew (Fig. S1), and then incubated in the same climate chamber for another 7 days. Three seedling tubes were used for each treatment, and the experiment was repeated three times.

Symptoms in tomato seedlings were visually scored using a disease scale of 0 to 2, as described by Marian *et al.* (2018), where 0=no symptoms, 1=small areas of the hypocotyl showing necrosis, and 2=a wilted seedling or large areas of the seedling showing necrosis. Disease severity was assessed using the following formula:

Disease severity=(Σ [number of diseased seedlings in each disease scale×disease scale]/[total number of seedlings investigated×the highest disease scale])×100%.

### Pot experiment

As described later, the L-arabinose treatment significantly reduced the severity of bacterial wilt in the aforementioned tomato seedling bioassay. Therefore, the suppressive effects of the soil L-arabinose treatment on tomato bacterial wilt were evaluated in a series of pot experiments under glasshouse conditions. In the first experiment, we compared the disease suppressive effects of three different concentrations of L-arabinose against bacterial wilt on tomato plants grown in pots. In the second experiment, the suppressive effects of L-arabinose were compared with those of L-histidine. A treatment with L-histidine was recently shown to suppress tomato bacterial wilt (Seo *et al.*, 2016).

Germinated tomato seeds were sown in plastic trays (Bee pot Y-49; Canelon Kakou) containing a commercial potting soil mix (Saika-ichiban; Ibigawa Kogyo) and grown in a glasshouse (natural light at 28–30°C) until the seedlings reached the three- to four-leaf stage. Tomato seedlings were then transplanted into vinyl pots (9‍ ‍cm in diameter) comprising three layers: top and bottom layers, each containing 150‍ ‍g of commercial potting soil mix, and a middle layer containing 20‍ ‍g of river sand.

In the first experiment, plants were treated with 30‍ ‍mL soil drench of 0.1, 0.25, or 0.5% (w/v) L-arabinose solution. Control plants were treated with an equal volume of SDW. One day after the drench treatment, all plants were drench-inoculated with 100‍ ‍mL of a washed cell suspension of VT0801 (approximately 3×10^7^ CFU mL^–1^ in 10‍ ‍mM MgCl_2_·6H_2_O solution) to obtain a final concentration of *ca.* 1×10^7^ CFU g^–1^ soil. In the second experiment, tomato plants were treated by soil drenching with 30‍ ‍mL of 0.5% (=*ca.* 33‍ ‍mM) L-arabinose or 10‍ ‍mM (=*ca.* 0.16%) L-histidine solution and challenge inoculated with VT0801 as described above. These inoculated plants were maintained in a glasshouse (natural light at 28–30°C). In the first experiment, each treatment included five plants, and the experiment was repeated five times. In contrast, each treatment had eight plants, and the experiment was repeated three times in the second experiment.

A disease assessment was performed 14 days post pathogen inoculation (dpi). The symptoms of tomato plants were visually scored on a disease scale of 0 to 4, as described by Roberts *et al.* (1988), where 0=no wilt symptom (healthy), 1=up to 25% of the leaves wilted, 2=26–50% of the leaves wilted, 3=51–75% of the leaves wilted, 4=76–100% of the leaves wilted. Disease severity was calculated using the following formula: Disease severity=([the number of diseased plants in each disease scale×disease scale]/[total number of plants investigated×the highest disease scale])×100%.

### Quantification of *R. pseudosolanacearum*

Tomato plants were treated with 0.5% (w/v) L-arabinose solution and challenged with strain VT0801 as described for the pot experiment. The population density of the pathogen in the rhizosphere and stem (approximately 1‍ ‍cm above the cotyledon node) of tomato plants was assessed at 6 and 10 dpi. Samples were obtained from five plants at each time point. Rhizosphere soil samples were suspended in SDW and gently shaken at 150 rpm at room temperature for 15‍ ‍min. They were then serially diluted with SDW. Stem samples (approximately 2‍ ‍cm in length) were surface-sterilized with 70% ethanol for 1‍ ‍min and air-dried for 5‍ ‍min in a laminar flow cabinet. These samples were placed into a sterile 15-ml tube containing five chromium steel beads (1/4 inch) and 3‍ ‍mL of SDW and then ground using a bead-beating grinder (FastPrep-24^TM^ 5G; MP Biomedicals) with the following settings: speed=6.5 m/s, time=60 s×2. After grinding, the homogenates were serially diluted with SDW. Two or three 10-‍μL droplets of each dilution of rhizosphere soil and stem homogenates were spot-inoculated onto square plates (96×15‍ ‍mm) containing M-SMSA medium (French *et al.*, 1995) and incubated at 30°C for 36 h. Typical colonies of *R. pseudosolanacearum* that appeared elevated and fluidal with a pink center were counted. The experiment was repeated three times. The population was calculated as an average of five plants and expressed as log CFU g^–1^ root fresh weight or log CFU g^–1^ stem fresh weight.

### Analysis of tomato defense-related gene expression using real-time reverse-transcription PCR (RT-PCR)

Tomato plants were drench treated with 0.5% (w/v) L-arabinose solution and then challenged with *R. pseudosolanacearum* as described for the pot experiment. As controls, tomato plants treated with SDW were challenged with the pathogen. All plants were maintained in a controlled environment chamber (28°C, 14-h light/10-h dark cycle). At 3 dpi, 0.2‍ ‍g of stems (1‍ ‍cm above the cotyledon node) was sampled from tomato plants to examine the expression of *PR-1a* and *GluA*, *GluB* and *Osmotin-like protein* (*OLP*), *LoxD*, and *Le4*, which are related to the salicylic acid (SA), ethylene (ET), jasmonic acid (JA), and abscisic acid (ABA) signaling pathways, respectively.

Samples were transferred to Lysing Matrix Tubes (MP Biomedicals) and powdered using a bead-beating grinder (FastPrep-24^TM^ 5G; MP Biomedicals). RNA extraction and real-time RT-PCR were performed as described previously with some modifications (Marian *et al.*, 2019). Briefly, total RNA was extracted from powdered samples using the cetyltrimethylammonium bromide method. In real-time RT-PCR, cDNA was synthesized from 300‍ ‍ng of total RNA using ReverTra Ace qPCR RT Master Mix with a gDNA Remover (Toyobo) following the manufacturer’s protocol. Real-time RT-PCR was performed with a total volume of 10‍ ‍μL containing 2.2‍ ‍μL of RNase-free water, 5‍ ‍μL of 2× SYBR Premix *EX Taq* II (T1i RNaseH Plus; Takara Bio), 2‍ ‍μL of the cDNA template, and 0.4‍ ‍μL of 10‍ ‍μM of each forward and reverse gene-specific primer in Thermal Cycler Dice® Real Time System II (Takara Bio) according to the manufacturer’s instructions. The gene-specific primers used in the present study are listed in Table S1 (Milling *et al.*, 2011; Aimé *et al.*, 2013; Martínez-Medina *et al.*, 2013). Transcript levels were normalized using the *β-tubulin* gene mRNA level as an internal standard. The expression levels of target genes were calculated using the 2^–ΔΔCT^ method (Livak and Schmittgen, 2001) and given as a value relative to untreated plants (not inoculated with L-arabinose and the pathogen). The real-time RT-PCR experiment was conducted once with nine biological replicates for each treatment and two technical repetitions for each replicate.

### Antibacterial activity of L-arabinose

The effects of L-arabinose on the proliferation of *R. pseudosolanacearum* VT0801 were assessed according to the method of Seo *et al.* (2016) with a slight modification. Briefly, 10‍ ‍μL of the cell suspension of VT0801 (approximately 9×10^7^ CFU mL^–1^) was inoculated into 10‍ ‍mL of CPG broth containing either 0.5% (w/v) L-arabinose or SDW as a control. After culturing at 30°C with shaking at 200 rpm for 18 h, bacterial growth was assessed by measuring the OD_600_ of the culture broth using a spectrophotometer. Three replicates were prepared for each treatment.

### Effects of L-arabinose on the pathogenicity of *R. pseudosolanacearum*

The effects of L-arabinose on the pathogenicity of *R. pseudosolanacearum* were examined by the method of Seo *et al.* (2016) with a slight modification. Strain VT0801 was cultured with or without 0.5% (w/v) L-arabinose in CPG broth medium at 30°C for 24 h with shaking at 200 rpm. Cell suspensions (approximately 3×10^7^ CFU mL^–1^) were prepared from each culture broth as described for the pot experiment. Three- and four-leaf stage tomato plants grown in pots were drench-inoculated with 100‍ ‍mL of the VT0801 cell suspension and maintained in the glasshouse for 14 days. Each treatment contained five plants with three replications.

### Chemotaxis of *R. pseudosolanacearum* towards L-arabinose

The chemotactic response of *R. pseudosolanacearum* towards L-arabinose was examined using the modified hard agar plug (tHAP) assay (Elgamoudi *et al.*, 2018) with a slight modification. In brief, an 8-mm hard agar plug (HAP) was made from a phosphate-buffer saline (PBS) agar (8‍ ‍g L^–1^, NaCl; 0.2‍ ‍g L^–1^, KCl; 1.44‍ ‍g L^–1^, Na_2_HPO_4_; 0.24‍ ‍g L^–1^, KH_2_PO_4_; 40‍ ‍g L^–1^ agar; pH 7.4) plate supplemented with 1% (w/v) triphenyltetrazolium chloride (TTC) and 0.5% (w/v) L-arabinose. HAP containing 0.5% (w/v) L-glutamine instead of L-arabinose served as a positive control (Hasegawa *et al.*, 2018), whereas that containing PBS served as a negative control. A cell suspension of VT0801 (approximately 5×10^8^ CFU mL^–1^ in PBS) was mixed at a 1:1 ratio with 0.6% (w/v) PBS agar (tempered at 50°C) and quickly poured into Petri dishes (90×15‍ ‍mm) containing HAP (the plug was placed 1.5‍ ‍cm from the edge of the plate). Plates were left to cool for 10‍ ‍min and then incubated at 30°C for 24 h. Plates were prepared as three replicates. Chemotactic activity was detected by the appearance of a red zone (colorless TTC was converted to insoluble and red 1,3,5-triphenylformazan by migrated bacteria) around HAP. To count migrated bacteria, HAP were recovered from three replicate plates and transferred into 4‍ ‍mL of PBS, vortexed, and incubated at room temperature for 30‍ ‍min. PBS was then serially diluted and spot-inoculated onto M-SMSA, as described, for the quantification of *R. pseudosolanacearum*. The population of VT0801 was calculated as log CFU mL^–1^ PBS. The experiment was repeated three times.

### Statistical analysis

Differences among the treatments in the tomato seedling bioassay and pot experiments were analyzed using Tukey’s test (*P*<0.05). Data on gene expression were analyzed using the Student’s *t*-test (*P*<0.05). Bacterial counts in the pot experiment and chemotaxis assay were transformed into logarithmic values and analyzed using the Student’s *t*-test or Tukey’s test (*P*<0.05). The optical densities (OD_600_) of bacterial cultures were compared using the Student’s *t*-test (*P*<0.05). All analyses were performed using the BellCurve for Excel (version 2.13; Social Survey Research Information).

## Results

### Sugar assimilation by *R. pseudosolanacearum*

In order to screen sugars that are not assimilated by *R.‍ ‍pseudosolanacearum*, strain VT0801 was grown in M63‍ ‍minimal medium containing L-arabinose, D-fructose, D-galactose, D-glucose, maltose, D-mannose, D-raffinose, D-ribose, sucrose, or D-xylose as the sole carbon source. The results obtained showed that strain VT0801 did not multiply in medium containing L-arabinose, maltose, D-raffinose, or D-ribose (Table 1), indicating that *R. pseudosolanacearum* is unable to assimilate these sugars for its growth. Based on this result, these four sugars were selected for the subsequent tomato seedling bioassay.

### Effects of selected sugars on tomato bacterial wilt in the tomato seedling bioassay

Tomato seedlings grown for 3 days in sterile vermiculite amended with L-arabinose, maltose, D-raffinose, or D-ribose were challenged with strain VT0801. In the control treatment, tomato seedlings were heavily infected with the pathogen, and disease severity reached *ca.* 72% at 7 dpi (Table 2). On the other hand, seedlings in all sugar treatments showed milder symptoms than those in the control treatment. Notably, the L-arabinose treatment significantly reduced disease severity to *ca.* 38%, implying that a soil treatment with L-arabinose has the capacity to suppress tomato bacterial wilt. Therefore, L-arabinose was selected as a candidate sugar and subjected to pot experiments under glasshouse conditions.

### Efficacy of L-arabinose under glasshouse conditions

**Fig. 1.**
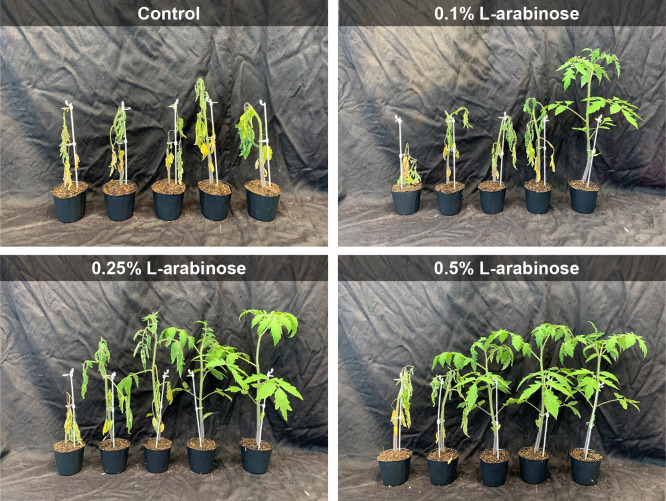
Development of bacterial wilt symptoms on tomato plants drench-treated with 0.1, 0.25, and 0.5% L-arabinose. Photos were obtained 14 days after the *Ralstonia pseudosolanacearum* inoculation.

In the first pot experiment, 0.1, 0.25, and 0.5% L-arabinose were applied to tomato seedlings grown in pots to examine their suppressive effects on tomato bacterial wilt under glasshouse conditions. In the control treatment, seedlings were intensively infected with the pathogen and disease severity reached 96% at 14 dpi (Fig. 1 and Table 3). In contrast, the drench treatment with L-arabinose suppressed bacterial wilt (Fig. 1). The suppressive effects of L-arabinose slightly increased in a dose-dependent manner; however, significant differences were observed among L-arabinose treatments (Table 3). The drench treatment with 0.5% L-arabinose reduced disease severity by *ca.* 87% from that with the control treatment, followed by 0.25% L-arabinose (*ca.* 70%) and 0.1% L-arabinose (*ca.* 48%).

In the second pot experiment, the wilt suppressive effects of 0.5% (*ca.* 33‍ ‍mM) L-arabinose were compared with those of 10‍ ‍mM (*ca.* 0.16%) L-histidine. Both treatments significantly reduced disease severity from that with the control treatment (Table 4). Comparisons of the L-arabinose and L-histidine treatments showed that the suppressive effects of the former were significantly stronger than those of the latter.

### Quantification of *R. pseudosolanacearum*

**Fig. 2.**
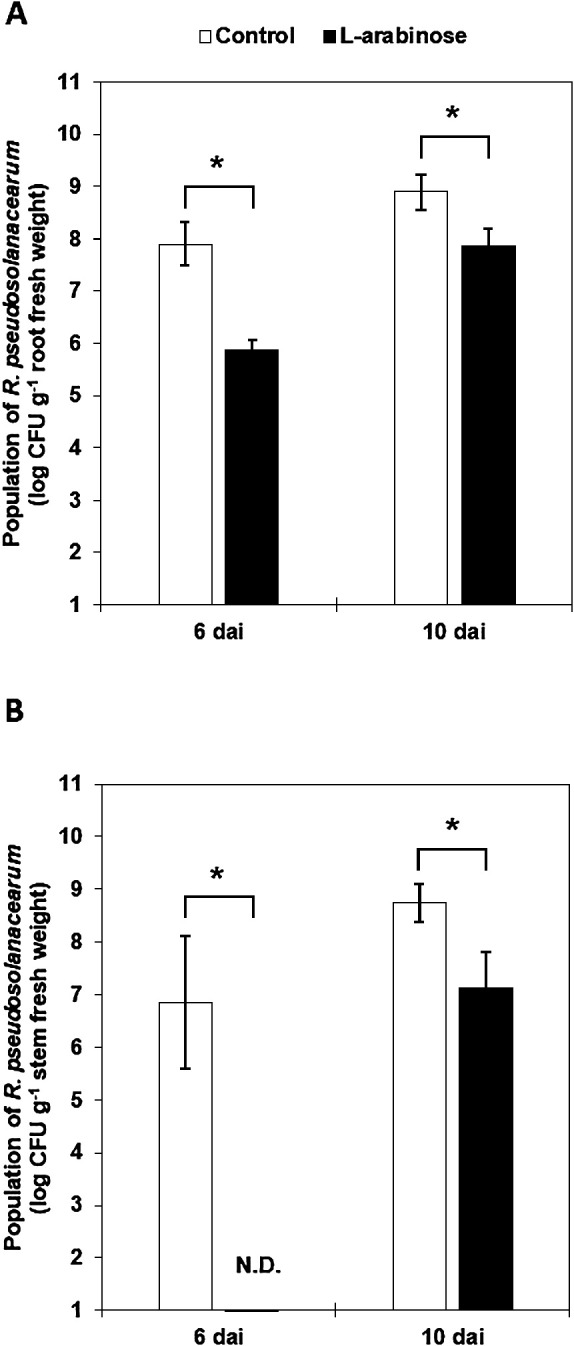
Population of *Ralstonia pseudosolanacearum* in the rhizosphere (A) and stem (B) of tomato plants treated with 0.5% L-arabinose. Samples were obtained 6 and 10 days after pathogen inoculation (dpi). N.D., not detected. Bars represent the mean±SD of three independent experiments. The asterisk indicates a significant difference between treatments according to the Student’s *t*-test at *P*<0.05.

The effects of the L-arabinose treatment on the proliferation of *R. pseudosolanacearum* in the rhizosphere and stem of tomato plants were investigated at 6 and 10 dpi. The pathogen population in the rhizosphere was significantly lower in L-arabinose-treated plants (5.7 and 7.9 log CFU g^–1^ root fresh weight) than in control plants (7.9 and 8.9 log CFU g^–1^ root fresh weight) at 6 and 10 dpi, respectively (Fig. 2A). Similarly, the pathogen population in the stems of L-arabinose-treated plants was maintained at a lower level than in the control plants (Fig. 2B). The pathogen was not detected or was present at a significantly lower level in the stems of L-arabinose-treated plants (7.1 log CFU g^–1^ stem fresh weight) than in those of control plants (6.7 and 8.7 log CFU g^–1^ stem fresh weight) at 6 and 10 dpi, respectively. These results indicated that the drench treatment with L-arabinose directly or indirectly suppressed pathogen proliferation in the rhizosphere and stem of tomato plants.

### Induction of defense-related genes in tomato by the L-arabinose treatment

**Fig. 3.**
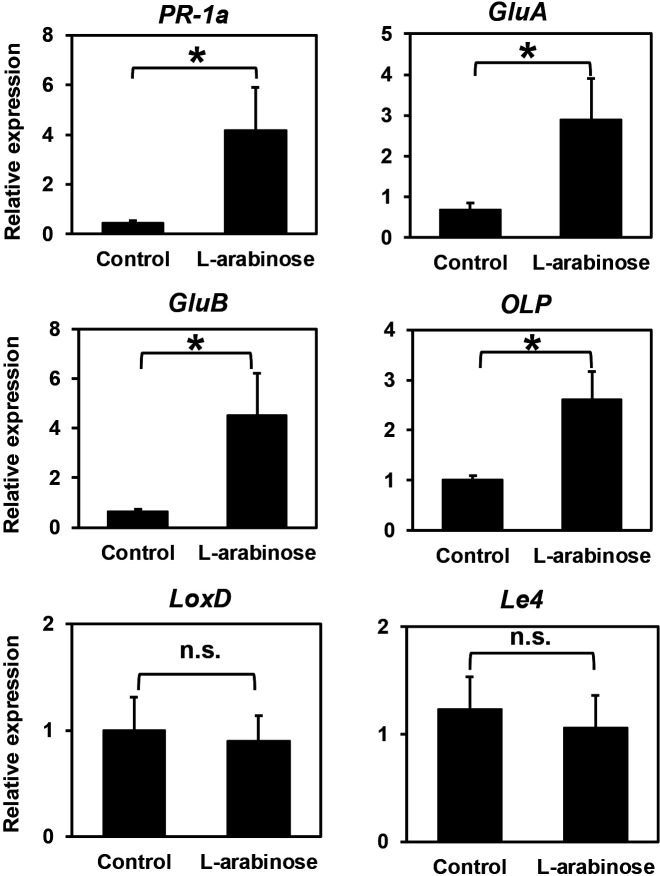
Relative expression of defense-related genes in stems of SDW- and L-arabinose-treated tomato plants 3 days after the pathogen inoculation. Expression was measured using quantitative real-time RT-PCR and normalized to the housekeeping gene *β-tubulin*. Values represent the mean±standard error in reference to the untreated uninoculated control. The asterisk indicates a significant difference between treatments according to the Student’s *t*-test at *P*<0.05, and n.s. indicates not significant.

The expression of six defense-related genes in the stems of control and L-arabinose-treated tomato plants was assessed using real-time RT-PCR at 3 dpi. The expression of the SA-responsive genes, *PR-1a* and *GluA*, was significantly stronger in L-arabinose-treated plants than in control plants (Fig. 3). Moreover, the expression of ET-responsive genes, *GluB* and *OLP*, was significantly stronger in L-arabinose-treated plants than in control plants. In contrast, the JA-responsive gene *LoxD* and ABA-responsive gene *Le4* were not induced following either treatment.

### Antibacterial activity of L-arabinose

Strain VT0801 was cultured in CPG broth amended with L-arabinose for 18 h. The results obtained showed that the cell density of VT0801 in L-arabinose-amended broth increased to a similar level to that in control broth (Fig. S2), indicating that L-arabinose does not exhibit antibacterial activity against *R. pseudosolanacearum*.

### Effects of L-arabinose on the pathogenicity of *R. pseudosolanacearum*

As shown in Fig. S3, the disease severity of tomato plants inoculated with strain VT0801 cultured in CPG broth containing L-arabinose was not significantly different from that of plants inoculated with the strain cultured in CPG broth. This result suggested that the pathogenicity of strain VT0801 was not affected by the pretreatment with L-arabinose.

### Chemotactic response to L-arabinose

The chemotactic response of VT0801 to L-arabinose was assessed using the tHAP assay. As shown in Fig. S4A, the red zone, an indication of bacterial cell accumulation, appeared around the plug containing L-glutamine (positive control), but not around those containing L-arabinose and PBS (negative control). Quantitative measurements showed that the number of migrated VT0801 cells was significantly higher around the plug containing L-glutamine than around those containing L-arabinose and PBS (Fig. S4B). Moreover, the cell number around the plug containing L-arabinose was not significantly different from that around the PBS plug. These results demonstrated that *R. pseudosolanacearum* did not exhibit a chemotactic response to L-arabinose.

## Discussion

In the present study, we demonstrated that the application of four sugars (L-arabinose, maltose, D-raffinose, and ribose), which are not assimilated by *R. pseudosolanacearum* strain VT0801 (Table 1), decreased the severity of bacterial wilt in tomato seedlings to varying degrees in the tomato seedling bioassay (Table 2). L-arabinose exerted significantly stronger disease suppressive effects than the other three sugars. Soil drenching with 0.1% to 0.5% L-arabinose achieved significant protection against tomato bacterial wilt under a very high pathogen pressure (10^7^ CFU g^–1^ soil) in the greenhouse pot experiments. The suppressive effects of L-arabinose slightly increased at higher concentrations (Fig. 1 and Table 3). Moreover, the wilt suppressive effects of the 0.5% (*ca.* 33‍ ‍mM) L-arabinose treatment were significantly stronger than those of 10‍ ‍mM (*ca.* 0.16%) L-histidine (Table 4), which was previously identified as an effective bacterial wilt inhibitory compound (Seo *et al.*, 2016), suggesting that L-arabinose has potential as an inhibitory compound against tomato bacterial wilt. The efficacy of L-histidine in the present study appeared to be lower than that achieved in a previous study by Seo *et al.* (2016). This may be due to differences in the bacterial strains used and/or experimental conditions. Previous studies reported the *in vitro* and* in planta* suppressive effects of natural organic compounds against various plant pathogens, including bacterial wilt pathogen (Posas and Toyota, 2010; Llorens *et al.*, 2017; Kim *et al.*, 2018; Jiménez-Reyes *et al.*, 2019; Jamiołkowska, 2020). However, to the best of our knowledge, this is the first study to report the control of tomato bacterial wilt by L-arabinose. The ability of RSSC to assimilate sugars including L-arabinose has been shown to vary at the strain level (Horita *et al.*, 2005). Therefore, further research is required to investigate the protective effects of L-arabinose against bacterial wilt caused by L-arabinose-assimilating strains.

Posas *et al.* (2007) also reported that the application of certain types of amino acids and glucose to field soil markedly suppressed tomato bacterial wilt. They indicated that disease suppression was due to soil microbial activity being enhanced by the addition of organic compounds. In contrast, as described above, L-arabinose suppressed bacterial wilt in the tomato seedling bioassay performed under axenic conditions, suggesting that mechanisms other than the stimulation of soil microbial activity are involved in the suppressive effects of L-arabinose. Since the bacterial wilt pathogen initially colonizes the host rhizosphere, then invades roots, and finally reaches the stems (Wei *et al.*, 2018), we investigated the effects of the L-arabinose treatment on pathogen proliferation in the rhizosphere and stems, and found that pathogen populations in the rhizosphere and stem tissues of L-arabinose-treated plants were maintained at significantly lower levels than those of control plants for at least 10 dpi (Fig. 2). Nishiyama *et al.* (1999) reported that the pathogen population in stems correlated with that in rhizosphere soil. Therefore, the decreased pathogen population in the stem tissues of L-arabinose-treated plants may be a consequence of the suppression of pathogen proliferation in the rhizosphere. Chemotaxis towards compounds present in the root exudates of host plants is an essential initial step in rhizosphere colonization by soil-borne bacterial pathogens (Yao and Allen, 2006; Matilla and Krell, 2018). Therefore, we speculated that the application of chemoattractants to soil may disturb the migration of a pathogen to the roots, thereby suppressing colonization of the rhizosphere by the pathogen. However, *R. pseudosolanacearum* strain VT0801 did not show a chemotactic response towards L-arabinose (Fig. S4), implying that the reduced pathogen population in the rhizosphere was not attributed to the disturbance of its migration to the roots by L-arabinose. Furthermore, the results from the *in vitro* antibacterial and pathogenicity assays (Fig. 2S and 3S) indicated that reductions in pathogen populations in the rhizosphere were not due to the antibacterial activity or pathogenicity-attenuating effects of L-arabinose. However, we did not investigate the impact of continuous exposure to L-arabinose on the behavior and virulence gene expression of the pathogen in the rhizosphere environment. Therefore, further studies are needed to verify that the direct effects of L-arabinose are not involved in the suppression of pathogen proliferation.

To protect against pathogen attack, plants have evolved sophisticated defense systems that recognize endogenous molecules released upon pathogen infection, known as damage-associated molecular patterns (DAMPs), and activate defense responses (Hou *et al.*, 2019). To date, many molecules, such as cellooligomers, xyloglucan polysaccharides, sucrose, and glucose, have been identified as DAMPs. L-arabinose is a major carbohydrate component of plant cell wall polysaccharides (Kotake *et al.*, 2016). Therefore, L-arabinose may be recognized as a DAMP by tomato plants, triggering defense responses. In the present study, the accumulation of transcripts of the SA-dependent defense genes, *PR-1a* and *GluA*, and ET-dependent defense genes, *GluB* and *OLP*, was significantly greater in the stem tissues of L-arabinose-treated plants upon the pathogen inoculation than in those of pathogen-inoculated control plants (Fig. 3). This result suggests that the drench treatment with L-arabinose elicited SA- and ET-dependent defenses in tomato plants. Previous studies indicated that SA and ET signaling pathways play a significant role in the defense of resistant tomato cultivars against bacterial wilt pathogens (Milling *et al.*, 2011; Takahashi *et al.*, 2014; Baichoo and Jaufeerally-Fakim, 2017). Therefore, we will investigate whether SA- and ET-dependent defense responses are involved in the bacterial wilt suppression induced by L-arabinose in future studies.

In conclusion, the results of the present study clearly demonstrated that soil drenching with L-arabinose effectively suppressed tomato bacterial wilt and may contribute to the development of a safe, eco-friendly, and cost-effective product for the control of bacterial wilt. Further research is needed to obtain a more detailed understanding of the mechanisms underlying disease suppression by this compound. Additionally, the efficacy of L-arabinose under field conditions needs to be verified.

## Citation

Fu, H.-Z., Marian, M., Enomoto, T., Suga, H., and Shimizu, M. (2020) Potential Use of L-arabinose for the Control of Tomato Bacterial Wilt. *Microbes Environ ***35**: ME20106.

https://doi.org/10.1264/jsme2.ME20106

## Supplementary Material

Supplementary Material

## Figures and Tables

**Table 1.** *In vitro* sugar assimilation capacity of *Ralstonia pseudosolanacearum* strain VT0801

Sugars	Bacterial growth (OD_600_)^§^	Assimilation^£^
SDW (control)	0.003±0.003	
L-arabinose	0.005±0.003	–
D-fructose	0.039±0.004	+
D-galactose	0.741±0.018	+
D-glucose	0.748±0.020	+
Maltose	0.007±0.002	–
D-mannose	0.017±0.003	+
D-raffinose	0.006±0.003	–
D-ribose	0.007±0.002	–
Sucrose	0.716±0.046	+
D-xylose	0.013±0.002	+

^§^ Data represent the mean±SD of three replicates. ^£^ “+” indicates a positive assimilation, “–” indicates a negative assimilation.

**Table 2.** Effects of selected sugars on the severity of bacterial wilt in the tomato seedling bioassay

Treatments	Disease severity^§^
SDW (control)	72.2±10.1a
L-arabinose	37.8±8.4 b
Maltose	51.1±3.8 ab
D-raffinose	63.3±20.0 ab
D-ribose	51.1±10.2 ab

^§^ Disease severity=([the number of diseased plants in each scale×disease scale]/[total number of plants investigated×the highest disease scale])×100%. Data represent the mean±SD of three replicates. Different letters indicate significant differences among treatments according to Tukey’s test at *P*<0.05.

**Table 3.** Effects of soil drenching with L-arabinose at 0.1, 0.25, and 0.5% on the severity of tomato bacterial wilt assessed using the pot experiment

Treatments	Disease severity^§^
SDW (control)	96.0±8.9 a^£^
0.1% L-arabinose	50.0±35.1 b
0.25% L-arabinose	29.0±27.5 b
0.5% L-arabinose	13.0±18.6 b

^§^ Disease severity=([the number of diseased plants in each disease scale×disease scale]/[total number of plants investigated×the highest disease scale])×100%. ^£^ Each value represents the mean±SD of five independent experiments. Different letters indicate significant differences (*P*<0.05) among the treatments, according to Tukey’s test.

**Table 4.** Comparison of suppressive effects of L-arabinose and L-histidine on tomato bacterial wilt

Treatments	Disease severity ^§^
SDW (control)	100.0±0.0 a^£^
0.5% (*ca.* 33‍ ‍mM) L-arabinose	41.7±7.2 c
10‍ ‍mM (*ca.* 0.16%) L-histidine	75.0±12.5 b

^§^ Disease severity=[(the number of diseased plants in each disease scale×disease scale)/(total number of plants investigated×the highest disease scale)]×100%. ^£^ Each value represents the mean±SD of three independent experiments. Different letters indicate significant differences among the treatments according to Tukey’s test at *P*<0.05.
