# Supplementary material for: Potential Use of L-arabinose for the Control of Tomato Bacterial Wilt
Source: Microbes Environ. 2020 Oct 22;35(4):ME20106. doi: 10.1264/jsme2.ME20106 (PMC7734405; doi:10.1264/jsme2.ME20106)
Supplement: Supplementary file 1 — Supplementary Material [file 35_20106_s1.pdf]

Table S1: Primers used in real-time RT-PCR analysis of tomato defense-related gene expression

Target gene	Primer sequence (5′–3′) [§]	References
PR-1a (pathogenesis-related protein-1a)	F-TCTTGTGAGGCCCAAAATTC<br>R-ATAGTCTGGCCTCTCGGACA	Aimé *et al.* (2013)
GluA (acidic extracellular β-1,3-glucanase)	F-GGTCTCAACCGCGACATATT<br>R-CACAAGGGCATCGAAAAGAT	Aimé *et al.* (2013)
GluB (basic intracellular β-1,3-glucanase)	F-TCTTGCCCCATTTCAAGTTC<br>R-TGCACGTGTATCCCTCAAAA	Aimé *et al.* (2013)
Osmotin-like protein	F-TGTACCACGTTTGGAGGACA<br>R-ACCAGGGCAAGTAAATGTGC	Milling *et al.* (2011)
LoxD (lipoxygenase D)	F-CCTGAAATCTATGGCCCTCA<br>R-ATGGGCTTAAGTGTGCCAAC	Aimé *et al.* (2013)
Le4 (desiccation protective protein)	F-ACTCAAGGCATGGGTACTGG<br>R-CCTTCTTTCTCCTCCCACCT	Martínez-Medina *et al.* (2013)
β-Tubulin	F-AACCTCCATTCAGGAGATGTTT<br>R-TCTGCTGTAGCATCCTGGTATT	Aimé *et al.* (2013)

[§] F, forward primer; R, reverse primer.

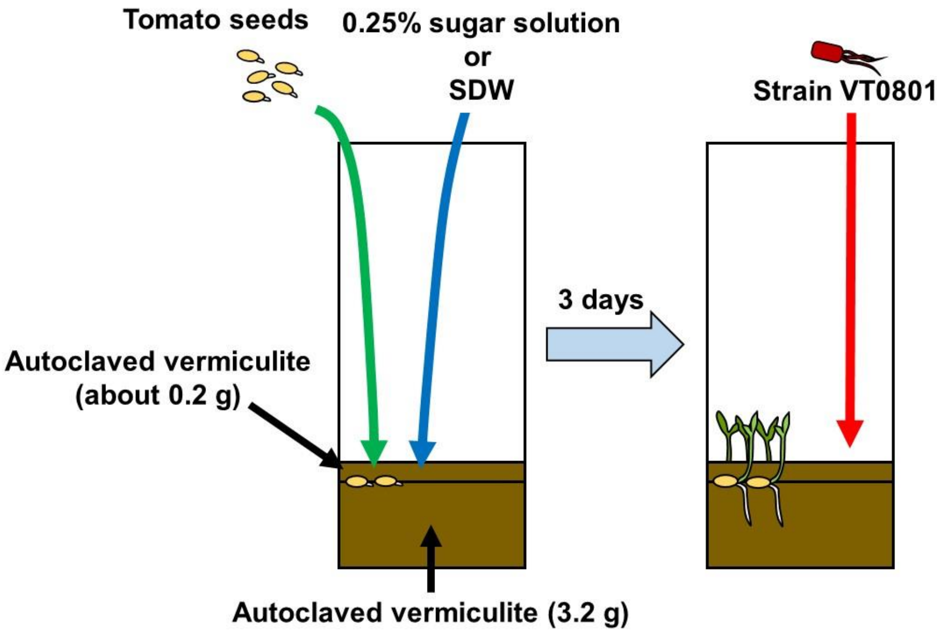

Fig. S1. Diagram of the experimental procedure for tomato seedling bioassay.

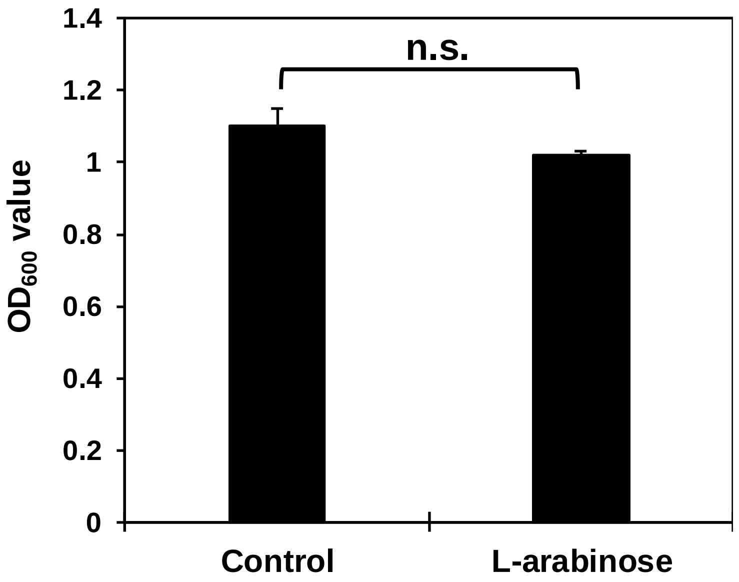

Fig. S2. Effect of L-arabinose on the *in vitro* multiplication of *Ralstonia pseudosolanacearum* strain VT0801. Bars represent the mean ± SD of three replications. n.s., not significant ($P < 0.05$, Student's t-test).

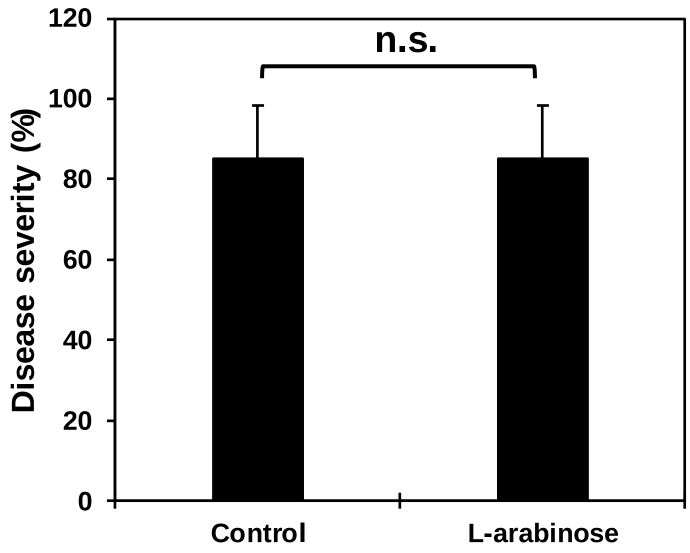

Fig. S3. Effect of L-arabinose on the pathogenicity of *Ralstonia pseudosolanacearum* strain VT0801. Bars represent the mean ± SD of three repeated experiments. n.s., not significant ($P < 0.05$, Student's *t*-test).

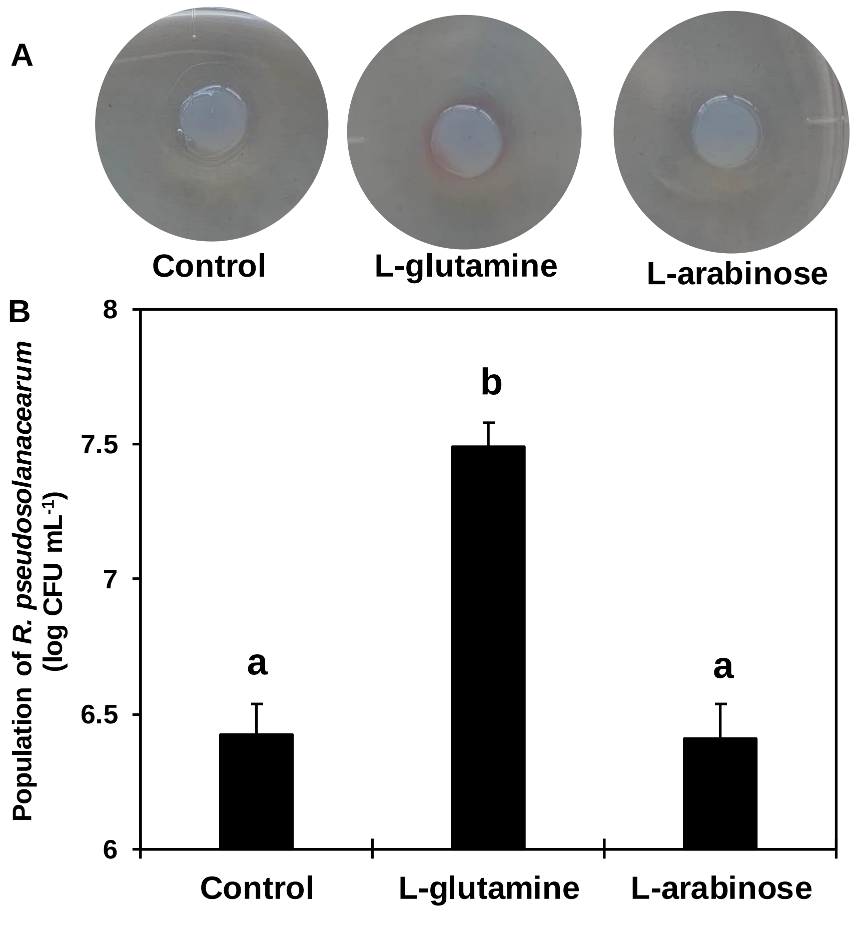

Fig. S4. Chemotactic response of *Ralstonia pseudosolanacearum* strain VT0801 to L-arabinose. A: Chemotactic response of VT0801 to phosphate buffered saline (PBS), 0.5% D-glutamine (positive control), and 0.5% L-arabinose on agar plugs. B: Population of bacteria migrated to the tested surface of agar plug. Bars represent the mean ± SD of three repeated experiments. Different letters above the bars indicate significant differences between treatments ($P < 0.05$, Tukey's test).
